# Characteristics of gliomas in patients with somatic IDH mosaicism

**DOI:** 10.1186/s40478-016-0302-y

**Published:** 2016-03-31

**Authors:** Charlotte Bonnet, Laure Thomas, Dimitri Psimaras, Franck Bielle, Elodie Vauléon, Hugues Loiseau, Stéphanie Cartalat-Carel, David Meyronet, Caroline Dehais, Jérôme Honnorat, Marc Sanson, François Ducray

**Affiliations:** Hospices Civils de Lyon, Hôpital Neurologique, Service de Neuro-oncologie, 59 Bvd Pinel, 69394 Lyon, Cedex France; Université Claude Bernard Lyon 1, Lyon, France; Department of Cancer Cell Plasticity, Cancer Research Centre of Lyon, INSERM U1052, CNRS UMR5286, Lyon, France; AP-HP, Groupe Hospitalier Pitié-Salpêtrière, Service de neurologie 2-Mazarin, Paris, France; AP-HP, Hôpitaux Universitaires Pitié Salpêtrière - Charles Foix, Laboratoire de Neuropathologie R. Escourolle, Paris, France; Sorbonne Universités, UPMC Univ Paris 06, Centre de recherche de l’Institut de Cerveau et de la Moelle Epinière (CRICM), UMR 975, Paris, France; INSERM U975, Paris, France; CNRS, UMR 7225, Paris, France; Centre Eugène Marquis, Medical Oncology, Rennes, France; Université de Bordeaux, Neurosurgery Department GH Pellegrin, Bordeaux, France; Hospices Civils de Lyon, Groupe Hospitalier Est, Service de Neuropathologie, Lyon, France; Lyon’s Neurosciences Research Center (CRNL) INSERM U1028, CNRS UMR5292, Lyon, France

**Keywords:** IDH mutation, Glioma, Somatic mosaicism, Ollier, Maffucci

## Abstract

*IDH* mutations are found in the majority of adult, diffuse, low-grade and anaplastic gliomas and are also frequently found in cartilaginous tumors. Ollier disease and Maffucci syndrome are two enchondromatosis syndromes characterized by the development of multiple benign cartilaginous tumors due to post-zygotic acquisition of *IDH* mutations. In addition to skeletal tumors, enchondromatosis patients sometimes develop gliomas. The aim of the present study was to determine whether gliomas in enchondromatosis patients might also result from somatic *IDH* mosaicism and whether their characteristics are similar to those of sporadic *IDH*-mutated gliomas. For this purpose, we analyzed the characteristics of 6 newly diagnosed and 32 previously reported cases of enchondromatosis patients who developed gliomas and compared them to those of a consecutive series of 159 patients with sporadic *IDH*-mutated gliomas. As was the case with sporadic *IDH* mutated gliomas, enchondromatosis gliomas were frequently located in the frontal lobe (54 %) and consisted of diffuse low-grade (73 %) or anaplastic gliomas (21 %). However, they were diagnosed at an earlier age (25.6 years versus 44 years, *p* < 0.001) and were more frequently multicentric (32 % versus 1 %, *p* < 0.001) and more frequently located within the brainstem than sporadic *IDH* mutated gliomas (21 % versus 1 %, *p* < 0.001). Their molecular profile was characterized by *IDH* mutations and loss of ATRX expression. In two patients, the same *IDH* mutation was demonstrated in the glioma and in a cartilaginous tumor. In contrast to sporadic *IDH* mutated gliomas, no enchondromatosis glioma harbored a 1p/19q co-deletion (0/6 versus 59/123, *p* = 0.03). The characteristics of gliomas in patients with enchondromatosis suggest that these tumors, as cartilaginous tumors, result from somatic *IDH* mosaicism and that the timing of *IDH* mutation acquisition might affect the location and molecular characteristics of gliomas. Early acquisition of *IDH* mutations could shift gliomagenesis towards the brainstem thereby mimicking the regional preference of histone mutated gliomas.

**Electronic supplementary material:**

The online version of this article (doi:10.1186/s40478-016-0302-y) contains supplementary material, which is available to authorized users.

## Introduction

Mutations in the *IDH1* or *IDH2* genes are found in the majority of adult diffuse grade II and grade III gliomas and are considered as the earliest oncogenic event in these tumors [50]. These mutations result in the abnormal production of 2-hydroxyglutarate (2-HG) which is structurally similar to alpha-ketoglutarate. 2-HG competitively inhibits multiple alpha-ketoglutarate enzymes leading to histone and DNA hypermethylation, altered cell differentiation, activation of enzymes implicated in HIF degradation and PDGFRA overexpression [14, 26, 30, 47]. In addition to gliomas, *IDH* mutations are particularly frequent in cartilaginous tumors [2, 50]. Ollier disease and Maffucci syndrome are two rare non-hereditary enchondromatosis syndromes characterized by the development of multiple benign cartilaginous tumors (enchondromas). Enchondromas appear during childhood and may progress to chondrosarcomas in up to 30 % of cases [3, 36]. In Maffucci syndrome, enchondromas are associated with soft tissue hemangiomas. The analysis of *IDH* mutations in multiple cartilaginous tumors and non-neoplastic tissues from enchondromatosis patients led to the conclusion that these pathologies are due to early post-zygotic acquisition of *IDH* mutations, resulting in somatic mosaic mutations of *IDH1* or *IDH2* [3, 36]. In addition to skeletal tumors, enchondromatosis patients may develop other neoplasm including juvenile granulosa tumors, cholangiocarcinomas, pituitary adenomas, acute myeloid leukemia and gliomas. The aim of the present study was to determine whether gliomas in enchondromatosis patients might also result from somatic *IDH* mosaicism and whether their characteristics are similar to those of sporadic *IDH*-mutated gliomas. For this purpose, we analyzed the characteristics of 6 newly diagnosed and 32 previously reported cases of enchondromatosis patients who developed gliomas, and compared them to those of a consecutive series of 159 patients with sporadic *IDH*-mutated gliomas.

## Materials and methods

We retrospectively reviewed the medical and radiological records of 6 patients with Ollier disease (*n* = 5) or Maffucci syndrome (*n* = 1) who were referred to our neuro-oncology departments (Pitié-Salpêtière Hospital in Paris, Hospices Civils of Lyon, CHU of Bordeaux and Rennes) for the diagnosis of glioma, as well as 159 consecutive patients who were diagnosed with an *IDH1* or *IDH2* mutated sporadic glioma between 2010 and 2014 (Hospices Civils of Lyon). Multicentric gliomas were defined as multiple gliomas without connecting T2/FLAIR signal abnormality and distinguished from multifocal gliomas (i.e. multiple gliomas with connecting T2/FLAIR signal abnormality) [1]. *IDH* mutations and 1p/19q co-deletion status were determined based on DNA extracted from blood and FFPE tumor using a standard protocol (Qiagen, QIAmp DNA mini Kit). *IDH1* codon 132 and *IDH2* codon 172 were sequenced using the Sanger method with the following primers: IDH1-Forward: TGTGTTGAGATGGACGCCTATTTG; IDH1-Reverse: TGCCACCAACGACCAAGTC; IDH2-Forward: GCCCGGTCTGCCACAAAGTC and IDH2-Reverse: TTGGCAGACTCCAGAGCCCA, as previously reported [21]. The 1p/19q co-deletion was determined based on the loss of heterozygosity technique (LOH) using microsatellite polymorphism markers as previously described [18]. In 3 patients, 1.5 μg of DNA extracted from frozen tumor tissue was outsourced to Integragen Company for the determination of the genomic profile based on Illumina SNP arrays [22]. In enchondromatosis gliomas, immunohistochemistry was performed on 4 μm thick sections of formalin-fixed paraffin embedded blocks with a Ventana Benchmark XT Device. The following antibodies were used after antigen retrieval to assess the expression of ATRX (anti-ATRX, Sigma, polyclonal, dilution 1/400), IDH1R132H (anti-IDH1R132H, Dianova, clone H09, dilution 1/50) and TP53 (clone DO.7, Dako, dilution 1/200). Previously reported cases of patients with Ollier disease or Maffucci syndrome who developed gliomas were identified through PubMed searches from January 1970 until September 2015 using the terms “Ollier disease”, “Maffucci syndrome”, “glioma” and “brain tumor”. We retrieved all relevant articles and checked additional references quoted in these articles. Categorical comparisons were performed using Fisher’s exact test and a t-test was used for quantitative variables. The threshold for statistical significance was *p* = 0.05.

## Results

The characteristics of our 6 patients are presented in Table 1 and those of the 32 previously published patients are shown in Additional file 1: Table S1 [5–8, 11, 12, 15, 16, 19, 20, 23–25, 31–35, 37–40, 43–46, 48, 49]. The characteristics of all of the patients are summarized in Table 2.

**Table 1:** Clinical, histological and molecular characteristics of our 6 patients with enchondromatosis who developed glioma

	Age at glioma diagnosis (years)	Sex	Histology	*IDH* mutation	1p19q co-deletion	ATRX loss of expression	Location	Multi-centric	History of chondrosarcoma	Survival (years)
Ollier disease										
1	28	F	OAII	*IDH1* R132H	No	Yes	T (*n* = 1), Fr (*n* = 1)	Yes	No	2.5+
2	26	M	-	-	-	-	Fr (*n* = 2)	Yes	Yes	1+
3	30	F	OII	*IDH1* R132H	No	Yes	Fr (*n* = 1), T (*n* = 1)	Yes	No	4
4	31	M	GBM	*IDH1* R132H	No	Yes	Fr (*n* = 2), P (*n* = 1)	Yes	No	0.75
5	31	F	OAIII	*IDH1* R132H	No	Yes	Fr	No	Yes	1.5+
Maffucci syndrome										
6	30	M	OAII	*IDH2* R132S	No	Yes	B	No	No	3+

*M* male, *F* female, *OAII/III* grade II/III oligo-astrocytoma, *OII* grade II oligodendroglioma, *GBM* glioblastoma, −: data not available, *T* temporal, *P* parietal, *Fr* frontal, *B* brainstem, +: alive at last news

**Table 2:** Summary of the clinical, histological and molecular characteristics of Ollier disease (OD), Maffucci syndrome (MS), enchondromatosis (OD + MS) -present series and literature- and sporadic *IDH* mutated glioma patients

	Ollier disease	Maffucci syndrome	Enchondro-matosis	Sporadic *IDH* mutated gliomas	Enchondro-matosis versus sporadic IDH mutated glioma *P*-value
N	28	10	38	159	
N of gliomas	45	12	57	161	
Sex ratio (M/F)	18/10	7/3	25/13	91/68	0.4
Median age (years, range)	24.7 (6–46)	28.1 (17–39)	25.6 (6–46)	44 (6–81)	<0.0001
Histology					
Grade II	68 % (17/25)	87.5 % (7/8)	72.7 % (24/33)	47 % (75/159)	0.01
Grade III	24 % (6/25)	12.5 % (1/8)	21.2 % (7/33)	39 % (62/159)	0.05
Grade IV	8 % (2/25)		6.1 % (2/33)	14 % (22/159)	0.4
Location					
Frontal	53.4 % (24/45)	58 % (7/12)	54.1 % (31/57)	67,1 % (108/161)	0.1
Parietal	4.4 % (2/45)		3.6 % (2/57)	7.5 % (12/161)	0.4
Insular	4.4 % (2/45)		3.6 % (2/57)	5,6 % (9/161)	0.7
Temporal	13.3 % (6/45)		10.5 % (6/57)	11.8 % (19/161)	1
Occipital	2.2 % (1/45)		1.8 % (1/57)	1.9 % (3/161)	1
Brainstem	15.7 % (7/45)	42 % (5/12)	21 % (12/57)	0.6 % (1/161)	<0.0001
Gliomatosis	4.4 % (2/45)		3.6 % (2/57)	5 % (8/161)	1
Thalamic	2.2 % (1/45)		1.8 % (1/57)	0.6 % (1/161)	1
Multicentric	39.2 % (11/28)	10 % (1/10)	31.6 % (12/38)	1.3 % (2/159)	<0.0001
*IDH* mutation	83 % (5/6)	100 % (2/2)	87.5 % (7/8)	100 %	-
1p/19q co-deletion	0 % (0/5)	0 % (0/1)	0 % (0/6)	48 % (59/123)	0.03
Other intracranial Tumor	25 % (7/21)	43 % (3/7)	36 % (10/28)	1.9 % (3/159)	<0.0001
History of chondrosarcoma	12.5 % (3/24)	42.8 % (3/7)	19.3 % (6/31)	0 %	<0.0001

*M* male, *F* female

**Fig. 1:**
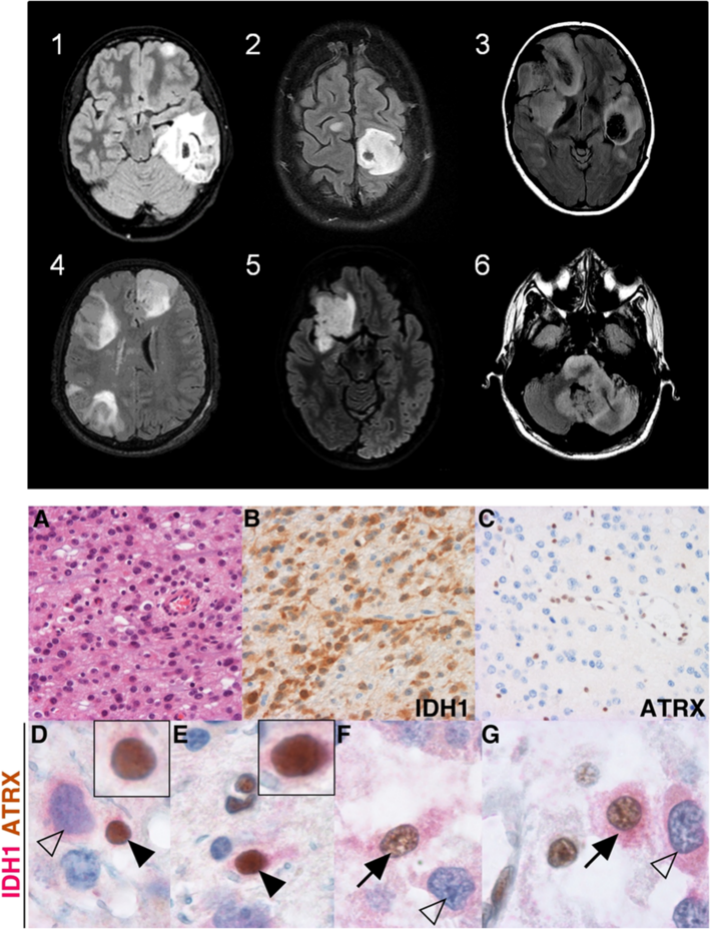
MRI characteristics of the diffuse gliomas in the 6 new enchondromatosis patients and double staining with anti-IDH1R132H and anti-ATRX antibodies. Top: MRI findings of patient 1 to patient 6 at diagnosis (axial T2/fluid-attenuated inversion recovery (FLAIR) sequences) demonstrating multicentric tumors in patients 1, 2, 3 and 4. Bottom: histological characteristics of the recurrent tumor of patient 3. **a** Hematoxylin eosin staining demonstrating an anaplastic glioma. **b** IDH1 R132H expression (brown signal). **c** Diffuse loss of ATRX expression in tumor cells and maintained expression (*brown signal*) in endothelial cells. **d**-**g**) Double staining demonstrating rare cells with a normal oligodendrocyte (*black arrowheads*) or astrocyte (*black arrows*) morphology expressing both IDH1 R132H (*red signal*) and ATRX (*brown signal*) (inserts showing same cells at higher magnification) and tumor cells with abnormal morphology and expressing IDH1 R132H but not ATRX (*white arrows*)

The diagnosis of Ollier disease (*n* = 28) or Maffucci syndrome (*n* = 10) was made during childhood based on the occurrence of multiple enchondromas predominating on the articulations of the knees, hands and toes. The median age at glioma diagnosis was 25.6 years. At this time, six patients (19 %) had a previous history of chondrosarcoma. Clinical presentation consisted of a variable association of seizures (38 %), progressive focal deficits (41 %) and intracranial hypertension (21 %). Radiological presentation was suggestive of diffuse glioma in all of the patients (Fig. 1). Tumors were most frequently located in the frontal lobe (54 %) and in the brainstem (21 %). Contrast enhancement was present in 27 % of cases. Twelve patients (32 %) had multicentric gliomas that were synchronous in eleven patients and metachronous in one patient. Multicentric gliomas were confined to the hemispheres in 8 patients and involved both the hemispheres and the brainstem in 4 patients. Two patients had gliomatosis cerebri. Ten patients (36 %) had another intracranial tumor at the time of glioma diagnosis (skull base enchondroma *n* = 7, pituitary adenoma *n* = 2, skull base chondrosarcoma *n* = 1).

The glioma was histologically proven in 33 patients. Among the 5 patients without histological diagnosis, 4 patients had a typical aspect of brainstem glioma consisting of an infiltrative intra-axial T2/FLAIR hypersignal without contrast-enhancement, and 1 patient had an aspect suggestive of hemispheric low-grade glioma that was managed with initial follow-up (patient 2, Fig. 1). Histology consisted of diffuse low-grade glioma in 24 patients (73 %), anaplastic glioma in 7 patients (21 %) and glioblastoma in 2 patients (6 %). An astrocytic (*n* = 22, 66 %) or oligo-astrocytic phenotype (*n* = 8, 25 %) was more frequently observed than a pure oligodendroglial phenotype (*n* = 3, 9 %). *IDH* mutations were assessed in 8 cases (based on sequencing in 6 patients and on immunohistochemistry only in 2 patients) and detected in the tumors of 7 patients (*IDH1* R132H *n* = 5, *IDH2* R172S *n* = 2). In the negative case, no expression of *IDH1* R132H was detected on immunohistochemistry, but other *IDH* mutations were not assessed [38]. In two patients (patient 5 and a previously reported patient [34]) the presence of an *IDH* mutation could be analyzed in both the glioma and a skeletal tumor and the same mutation (*IDH1* R132H in patient 5 and *IDH2* R172S in [34]) was identified in these tumors. Since none of our patients underwent a biopsy of more than one glioma, the analysis of *IDH* mutations in different gliomas from the same patient could not be performed. No 1p/19q co-deletion was identified in the 6 cases in which it was assessed (oligo-astrocytoma *n* = 4, oligodendroglioma *n* = 1, glioblastoma *n* = 1). TP53 expression was studied in 6 patients and found to be expressed in 5 patients. In an additional patient, sequencing demonstrated missenses TP53 mutations [34]. ATRX expression was studied in 5 patients. A homogeneous loss of expression was observed in 4 patients and a heterogeneous loss of expression in 1 patient (patient 3). Genomic profiles of the tumors were obtained for 2 patients; one patient had an isolated LOH of the 17p region covering the TP53 locus (patient 3), and the other patient had a LOH of chromosome 9 associated with partial losses of chromosomes 14q and 15q and a partial gain of chromosome 11q (patient 5). In patient 3, double staining with anti-ATRX and anti-*IDH1* R132H antibodies identified: (i) areas with maintained ATRX expression in tumor cells, and (ii) areas with diffuse loss of ATRX expression in tumors cells. In these last areas, some very rare *IDH1* R132H and ATRX positive cells with a normal morphology were observed (Fig. 1). Treatment consisted of surgical resection (46 %), radiotherapy (80 %) and or chemotherapy (42 %). The median survival after glioma diagnosis was 5 years.

**Fig. 2:**
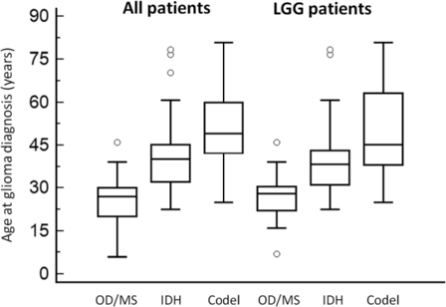
Age at glioma in patients with enchondromatosis and sporadic *IDH* mutated gliomas according to grade and 1p/19q co-deletion. Y axis: age at glioma diagnosis (years). X axis: OD/MS: enchondromatosis patients with glioma, IDH: patients with sporadic *IDH* mutated gliomas without 1p/19q co-deletion, Codel: patients with sporadic *IDH* mutated gliomas with 1p/19q co-deletion. Median age at glioma diagnosis, was lower in OD/MS than in IDH patients (25.6 vs 41 years, *p* < 0.001) and in IDH than in Codel patients (41 years vs 50.8 years, *p* < 0.001). The difference remained significant when taking into account only LGG (OD/MS vs IDH patients: 26.9 vs 39 years, *p* < 0.001; IDH vs Codel patients: 39 vs 49 years, *p* = 0.01)

Independent of histology and 1p/19q co-deletion status, gliomas in enchondromatosis patients were diagnosed at an earlier age than sporadic *IDH* mutated gliomas (25.6 years versus 44 years, *p* < 0.001, Fig. 2). In addition, they were more frequently multicentric (32 % versus 1 %, *p* < 0.001), more frequently involved the brainstem (21 % versus 1 %, *p* < 0.001) and were not associated with a 1p/19q co-deletion (0/6 versus 59/123, *p* = 0.03).

## Discussion

The high rate of multicentric gliomas, the identification of *IDH* mutations in all of the gliomas in which it could be fully assessed and the identical *IDH* mutations found in both the glioma and the skeletal tumor of two patients strongly suggest that, in most cases, similar to cartilaginous tumors, the development of gliomas in enchondromatosis patients results from somatic *IDH* mosaicism. The very rare *IDH1* R132H and ATRX positive cells with a normal morphology found in patient 3 may further support this hypothesis. Consistent with the demonstration of occasional *IDH* mutated cells in the normal bone, blood and bone marrow of some enchondromatosis patients, we speculate that these cells may correspond to normal glial cells with *IDH* mutations [3, 36]. However, this observation must be taken with caution since morphology is subjective and ATRX expression can be heterogeneous [41].

*IDH* mutations are considered to be the earliest oncogenic events in the majority of lower grade gliomas [10]. In animal models, *IDH* mutations have been shown to be sufficient to induce enchondromas and chondrosarcomas [17, 29]. To our knowledge, this demonstration has not been previously reported for gliomas, but the occurrence of gliomas in enchondromatosis patients, in whom *IDH* mutations are thought to occur as an early post-zygotic event suggests that *IDH* mutations can initiate gliomagenesis [3, 36]. In addition, the median age at glioma diagnosis in these patients (25 years) suggests that *IDH* driven gliomagenesis is a very slow process. In contrast to enchondromas, however, *IDH* mutation alone is probably not sufficient to induce gliomas. Additional alterations, such as *ATRX* and *TP53* mutations, are necessary [10]. Consistently, Moriya et al. identified a *TP53* mutation in the glioma but not in an enchondroma of their patient, though both lesions shared the same *IDH*2 mutation [34]. This could explain why enchondromas appear much earlier than gliomas in enchondromatosis patients.

It is unknown why only a small percentage of enchondromatosis patients (approximately 5 %) develop gliomas [45]. It could be related to a variable distribution and proportion of mutated cells within the brain, to the fact that only a small percentage of *IDH* mutated glial cells acquire additional alterations, or to the type of *IDH* mutation present. In cartilaginous tumors of enchondromatosis patients the *IDH1* R132C mutation is more frequent than the *IDH1* R132H mutation (70 and 15 %, respectively) [3, 36], while the *IDH1* R132H mutation is the most frequent mutation (90 %) in sporadic *IDH* mutated gliomas [50]. Enchondromatosis patients with an *IDH1* R132H mutation could have a higher risk of developing gliomas than patients with an *IDH1* R132C mutation. In our series, an *IDH1* R132H mutation was present in 5 out of the 7 enchondromatosis patients who developed diffuse glioma.

Not surprisingly, like sporadic *IDH* mutated gliomas, enchondromatosis gliomas were frequently located in the frontal lobe and were more frequently diffuse low-grade or anaplastic gliomas than glioblastomas. However, they differed from sporadic *IDH*-mutated gliomas in several aspects. First, they were diagnosed at an earlier age. This difference could be explained by the fact that in sporadic *IDH*-mutated gliomas, the *IDH* mutation is acquired later than in enchondromatosis patients. Second, enchondromatosis gliomas, compared to sporadic *IDH* mutated gliomas, more frequently involved the brainstem, which may also be related to an earlier origin of enchondromatosis gliomas (i.e., during the first years of life, which is a period associated with the development of infratentorial gliomas). Since both H3-K27M mutations (which are present in most cases of children brainstem gliomas) and *IDH* mutations alter histone methylation, brainstem gliomas may therefore require histone modification of precursor cells at an early stage of development [9, 28, 30]. This particularity could help explaining why early acquisition of *IDH* mutations mimics the regional preference of H3-K27M mutated gliomas and why gliomas in that location are rare in adults. At last, none of the 6 enchondromatosis gliomas that could be tested were associated with a 1p/19q co-deletion. This observation needs confirmation in a larger series, however, again it could be related to a different timing of oncogenesis because the 1p/19q co-deletion is virtually absent in pediatric gliomas [42]. Consistently, in the present series, as in previously reported series, patients with 1p/19q co-deleted gliomas were older at diagnosis than those with *IDH* mutated non 1p/19q co-deleted gliomas [13].

Finally, multicentric gliomas were much more frequent in enchondromatosis than in sporadic *IDH*-mutated gliomas. Multicentric gliomas (i.e., with no apparent continuity between tumors) account for 2 to 5 % of sporadic gliomas [1]. Their oncogenesis is debated. In some cases, evidence has been provided that the multiple tumors actually originated from the same clone [1]. However, the multicentric presentation of gliomas reported in patients with inherited glioma predisposition and the high incidence of systemic cancers reported in patients with multicentric gliomas also suggest that multicentric gliomas may originate from different clones in patients who have inherited genetic alterations predisposing them to gliomas [4, 27]. Our study shows that somatic mosaicism might be another mechanism leading to the development of multicentric gliomas. It remains to be determined whether somatic *IDH* mosaicism might be responsible for the occurrence of multicentric gliomas in patients without enchondromatosis.

## Conclusions

In addition to its retrospective design and its small sample-size, limitations of the present study include the limited number of patients in whom molecular characterization was possible, the absence of comprehensive molecular analysis and the absence of *IDH* mutation assessment in several gliomas from the same patient. Nevertheless, the analysis of glioma characteristics in patients with enchondromatosis sheds new light on *IDH* driven gliomagenesis. It provides evidence that the *IDH* mutation can initiate gliomagenesis and that the timing of *IDH* mutation acquisition might influence the location and molecular characteristics of gliomas.

## Additional file

Additional file 1: Table S1. The characteristics of those of the 32 previously published patients. (XLSX 15 kb)
